# Dark Adaptometry as a Diagnostic Tool in Retinal Diseases: Mechanisms and Clinical Utility

**DOI:** 10.3390/jcm14113742

**Published:** 2025-05-27

**Authors:** Anas Bakdalieh, Layth M. Khawaja, Minzhong Yu

**Affiliations:** 1 College of Medicine, Northeast Ohio Medical University, Rootstown, OH 44272, USA; abakdalieh@neomed.edu (A.B.);; 2 Department of Ophthalmology and Visual Sciences, University Hospitals Eye Institute, Case Western Reserve University, Cleveland, OH 44106, USA; 3 Cole Eye Institute, Cleveland Clinic Foundation, Cleveland, OH 44106, USA; 4 Department of Ophthalmology, Cleveland Clinic Lerner College of Medicine of Case Western Reserve University, Cleveland, OH 44195, USA

**Keywords:** dark adaptometry, rod–cone break, photopigment regeneration, age-related macular degeneration, retinitis pigmentosa, diabetic retinopathy, Stargardt disease, cone–rod dystrophy, vitamin A deficiency, congenital stationary night blindness

## Abstract

Dark adaptometry is a non-invasive functional test that assesses the retina’s ability to recover sensitivity in low-light conditions following photobleaching. This review explores the physiological mechanisms underlying dark adaptation (DA), including photopigment regeneration and the critical role of the retinal pigment epithelium in the visual cycle. We detail clinical protocols for dark adaptometry using modern instruments such as the AdaptDx, highlighting methodological advances that improve testing efficiency and reproducibility. The clinical utility of dark adaptometry is examined across a range of inherited and acquired retinal disorders, including age-related macular degeneration (AMD), retinitis pigmentosa (RP), Stargardt disease, diabetic retinopathy (DR), cone–rod dystrophy (CRD), vitamin A deficiency, and congenital stationary night blindness (CSNB). Dark adaptometry has emerged as a sensitive biomarker capable of detecting functional deficits before structural changes are evident, making it a valuable tool for early diagnosis and monitoring disease progression. However, limitations such as age-related variability, patient compliance, and lack of standardization remain challenges to broader clinical adoption. Continued refinement of dark adaptometry protocols and instrumentation is essential to maximize its diagnostic potential in ophthalmic practice.

## 1. Mechanism of Dark Adaptation

Dark adaptation (DA) is the physiological process by which the retina gradually restores its sensitivity to low-light conditions following exposure to bright illumination. This process is essential for visual performance in dim environments, such as entering a darkened room after being outdoors or driving into a tunnel on a sunny day. As adaptation progresses, the eye’s threshold for detecting light decreases, allowing for improved vision in the dark.

Clinically, DA is a valuable diagnostic tool used to assess the retina’s functional recovery after a controlled photobleaching event. The procedure involves exposing the eye to a standardized bright light to bleach a significant proportion of photopigments, followed by measuring the subject’s ability to detect incrementally dimmer stimuli over time. The rate of sensitivity recovery reflects the integrity of photoreceptor and retinal pigment epithelium (RPE) function.

The DA process is driven by the biochemical regeneration of visual pigments, particularly rhodopsin in rod photoreceptors. Upon exposure to light, rhodopsin is depleted, and its regeneration depends on the retinoid cycle within the RPE. As rhodopsin is restored, rod sensitivity improves, which is charted over time using a DA curve. A typical DA curve consists of two distinct phases: an initial rapid decline in threshold representing cone-mediated recovery, followed by a slower, prolonged phase reflecting rod-mediated recovery. The inflection point between these two phases, known as the rod–cone break, marks the transition from cone to rod dominance.

Dark adaptometry can be performed using chromatic stimuli to distinguish rod and cone contributions. Red light (~650 nm) preferentially stimulates cones, while blue–green light (~500 nm) targets rods. Deviations in the shape or timing of the DA curve, such as delayed rod–cone break or incomplete recovery, may indicate early dysfunction in diseases like age-related macular degeneration (AMD), retinitis pigmentosa (RP), or vitamin A deficiency.

### 1.1. Photopigment Regeneration

The regeneration of visual pigment is essential for DA and differs between rod and cone photoreceptors due to their distinct metabolic profiles and energy-handling capacities. Rods, which mediate scotopic vision, lack intrinsic glycogen stores and rely entirely on a continuous supply of glucose and oxygen from the choroidal circulation for their metabolic needs [1]. This dependency renders rods highly susceptible to hypoxia and metabolic dysregulation, particularly in conditions such as diabetic retinopathy (DR) or retinal vascular insufficiency. In contrast, cone photoreceptors, which support photopic vision, contain glycogen reserves and have a more robust capacity to buffer against transient metabolic stress [1].

These physiological differences between rods and cones are exploited in dark adaptometry, a functional test that measures the kinetics of visual pigment regeneration following photobleaching. Chromatic dark adaptometry employs wavelength-specific stimuli to isolate rod and cone responses: blue–green light (~500 nm) preferentially activates rods due to their peak spectral sensitivity, while red light (~650 nm) primarily stimulates cones [2]. By assessing the rate and extent of sensitivity recovery to these stimuli, clinicians can independently evaluate rod and cone function. Therefore, DA complements structural imaging and is particularly useful in diagnosing and monitoring retinal diseases such as AMD, RP, and DR.

### 1.2. Retinal Pigment Epithelium (RPE) and Visual Cycle

The RPE is a vital component of the outer blood-retina barrier, formed by tight junctions between adjacent RPE cells that establish a high-resistance interface. This barrier regulates the selective exchange of nutrients, ions, and metabolic waste products between the neural retina and the underlying choroidal circulation, maintaining retinal homeostasis. In addition to its barrier function, the RPE plays a central role in the visual cycle. It is responsible for the daily phagocytosis and degradation of shed photoreceptor outer segment discs, a process crucial for photoreceptor renewal. Furthermore, the RPE facilitates retinoid recycling: following phototransduction, all-trans-retinal is reduced to all-trans-retinol in photoreceptors and then transported to the RPE. There, it is enzymatically converted back to 11-*cis*-retinal through a series of reactions involving key proteins such as LRAT and RPE65. The regenerated 11-*cis*-retinal is then transported back to photoreceptors, where it recombines with opsin to regenerate rhodopsin, thereby sustaining continuous visual function [3].

The visual cycle is a tightly regulated, multi-step enzymatic process that regenerates 11-*cis*-retinal, the chromophore essential for phototransduction. This cycle involves coordinated activity across the photoreceptor outer segments, the interphotoreceptor matrix, and the RPE. Upon absorption of a photon, 11-*cis*-retinal bound to opsin is photoisomerized to all-trans-retinal, triggering activation of the visual pigment and initiating the phototransduction cascade. All-trans-retinal is then released from opsin and reduced to all-trans-retinol by photoreceptor-associated all-trans-retinol dehydrogenases (RDHs), such as RDH8, utilizing NADPH as a cofactor [3].

All-trans-retinol is transported from photoreceptors to the RPE via interphotoreceptor retinoid-binding protein (IRBP), which shields the hydrophobic retinoids in the aqueous interphotoreceptor matrix. Within the RPE cytoplasm, all-trans-retinol is esterified by lecithin retinol acyltransferase (LRAT) to form all-trans-retinyl esters. These esters serve as substrates for the key isomerohydrolase enzyme RPE65, which catalyzes the conversion of all-trans-retinyl esters to 11-*cis*-retinol. This isomerization is a crucial and energetically demanding step that requires iron as a cofactor and occurs within the smooth endoplasmic reticulum of the RPE.

Subsequently, 11-*cis*-retinol is oxidized to 11-*cis*-retinal by 11-*cis*-retinol dehydrogenases (e.g., RDH5, RDH11, or RDH10), using NAD+ as a cofactor. The regenerated 11-*cis*-retinal binds to cellular retinaldehyde-binding protein (CRALBP) to maintain its solubility and protect it from non-specific reactivity. It is then delivered back to the photoreceptors via IRBP, where it recombines with opsin to regenerate functional visual pigment such as rhodopsin (in rods) or cone opsins (in cones).

An important consideration in the visual cycle is whether complete biochemical recycling of all-trans-retinoid to 11-*cis*-retinal is required for full rhodopsin regeneration and DA. Lamb et al. [3] propose that total recycling is not always necessary, as photoreceptors can regain function even in the absence of complete retinoid turnover under certain conditions. Specifically, when the pool of retinoid precursors available for rapid mobilization to 11-*cis*-retinal exceeds the immediate demand of photoreceptors, full recycling may be bypassed or delayed without compromising visual performance. This buffering capacity may help maintain visual function during transient metabolic shifts.

However, in states of vitamin A deficiency or in pathological conditions that deplete retinoid reserves—such as malnutrition, liver disease, or genetic defects affecting the visual cycle enzymes—robust and efficient recycling of retinoids becomes critical for timely pigment regeneration and recovery of visual sensitivity. Under such circumstances, delayed or incomplete recycling can result in prolonged DA or even night blindness. Therefore, while full retinoid recycling may not always be strictly necessary under optimal conditions, its efficiency is essential for sustaining photoreceptor function during periods of metabolic stress or nutrient limitation.

### 1.3. Dynamics of Cone-to-Rod Transition

The cone critical duration, or the minimal duration to perceive a flash of light, increases to a maximum of 110 ms within the first 30 s of DA, while rod critical duration increases gradually to 210 ms after 20–30 min [4].

Following exposure to bright light, DA begins with rapid recovery of cone photoreceptor sensitivity, followed by slower, prolonged recovery of rod function [5]. This biphasic adaptation process reflects the distinct regenerative kinetics and thresholds of the two photoreceptor types. The relative contributions of rods and cones at any point during DA can be inferred by comparing sensitivity to stimuli of different wavelengths—typically 500 nm (favoring rod activation) and 650 nm (favoring cone activation). The differential response to these wavelengths helps determine which photoreceptor type is mediating visual perception at a given time point.

Cone photoreceptors recover their sensitivity relatively quickly, typically reaching a plateau within 5 to 10 min. In contrast, rods require a longer period—up to 40 min—to achieve maximal sensitivity in scotopic conditions. This sequential recovery is clearly depicted in DA curves, wherein the rod–cone break marks the transition point, usually around 5 min after light offset, when rods begin to dominate visual function [6].

In addition to threshold sensitivity, the temporal integration properties of rods and cones also evolve during adaptation. The critical duration—defined as the minimal flash duration required for light perception—increases with time spent in darkness. For cones, the critical duration rises rapidly to approximately 110 milliseconds within the first 30 s of DA. Rods, on the other hand, exhibit a gradual increase in critical duration, reaching up to 210 milliseconds after 20–30 min. Although this may appear counterintuitive, the increase in critical duration actually enhances light sensitivity: as photoreceptors adapt to darkness, they extend the time window over which they summate photons, allowing for the detection of dimmer stimuli. This temporal summation improves sensitivity at the expense of temporal resolution, forming a fundamental component of the DA process [4].

## 2. Clinical Protocols

The setup and assessment protocols for dark adaptometry, specifically using the AdaptDx (MacuLogix, Harrisburg, PA, USA) device, have been rigorously established to ensure accurate and reproducible evaluation of retinal function. This section outlines the standardized clinical protocol for dark adaptometry, emphasizing the setup process, the assessment procedure, and the key operational parameters involved in testing. These elements are critical for maintaining consistency in measurements and ensuring the clinical utility of DA as a diagnostic tool.

### 2.1. Setup

**Figure 1:**
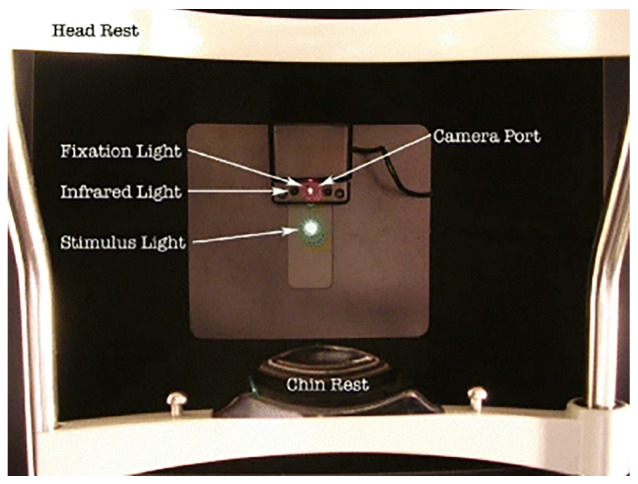
The patient’s view of the AdaptDx. The bleaching light is presented through an aperture co-localized with the stimulus light [7].

The setup for the AdaptDx device begins with preparing the patient in a controlled environment. The patient is positioned securely against a head and chin rest to ensure proper alignment and fixation (Figure 1). The test eye is dilated to achieve a pupil diameter of ≥6 mm, typically using a combination of 1% tropicamide and 2.5% phenylephrine. Adequate dilation is essential for consistent stimulus delivery and optimal measurement of retinal sensitivity. Prior to testing, corrective lenses are inserted into the device’s optical path to compensate for the patient’s refractive error at the standard 30 cm working distance. This ensures the accuracy of stimulus presentation and patient fixation. To isolate the test eye, the fellow eye is occluded with an eye patch, eliminating binocular interference during the measurement process.

The test begins with the presentation of a brief, standardized bleaching flash that photobleaches a significant portion of rhodopsin in the rod photoreceptors. This controlled desensitization simulates a transition from a photopic to a scotopic environment and initiates the DA process, during which the recovery of rod sensitivity is continuously monitored over time [7,8].

Once the patient is properly prepared, the test setup involves the use of an infrared camera to monitor the alignment of the patient’s test eye. The camera is positioned behind a fixation light and continuously displays an image of the test eye on a computer-based control screen. This setup allows the operator to visually confirm proper eye alignment in real time.

To facilitate precise alignment, the operator uses the reticule displayed on the infrared image of the eye to center the test eye on the red fixation light (635 nm). This step is critical for ensuring that both the bleaching flash and subsequent stimuli are consistently applied to the same retinal area throughout the test.

To initiate the DA process, the patient’s test eye is exposed to a photoflash (505 nm) that passes through a diffuser and a four-diameter aperture. This setup ensures that the entire targeted retinal area is uniformly bleached, thereby establishing a controlled baseline for subsequent sensitivity measurements.

Immediately following the bleaching photoflash, the testing procedure begins. The patient is instructed to focus on the fixation light, and sensitivity measurements are taken by presenting a series of stimulus lights at regular intervals. The patient is instructed to press a hand-held response button as soon as they notice the light. A response is considered valid if the button is pressed within 2 s of the stimulus being presented.

To isolate rod function from cone function, a 500 nm wavelength stimulus is used, which ensures accurate measurement of rod-mediated DA. The stimulus intensity is adjusted using a three-down/one-up staircase method, starting at an intensity of 5.00 cd/m^2^. Every 2 to 3 s, a 200 ms stimulus is presented. If the patient does not detect the stimulus, the intensity remains constant until a response is provided. Once the stimulus becomes detectable, the intensity is decreased by 0.3 log units for each successive presentation until the patient no longer detects the stimulus. At this point, the intensity is incrementally increased by 0.1 log units until the stimulus is once again detectable. This intensity measurement is then recorded as the threshold.

Typically, one threshold is recorded every minute during the protocol. The test concludes when either two consecutive threshold measurements are above 5 × 10^−3^ scot cd/m^2^, or when the 20 min testing window has elapsed—whichever comes first. The resulting measurements are plotted, and the Rod Intercept Time (RIT) is calculated via linear interpolation [7,8].

### 2.2. Assessment

**Figure 2:**
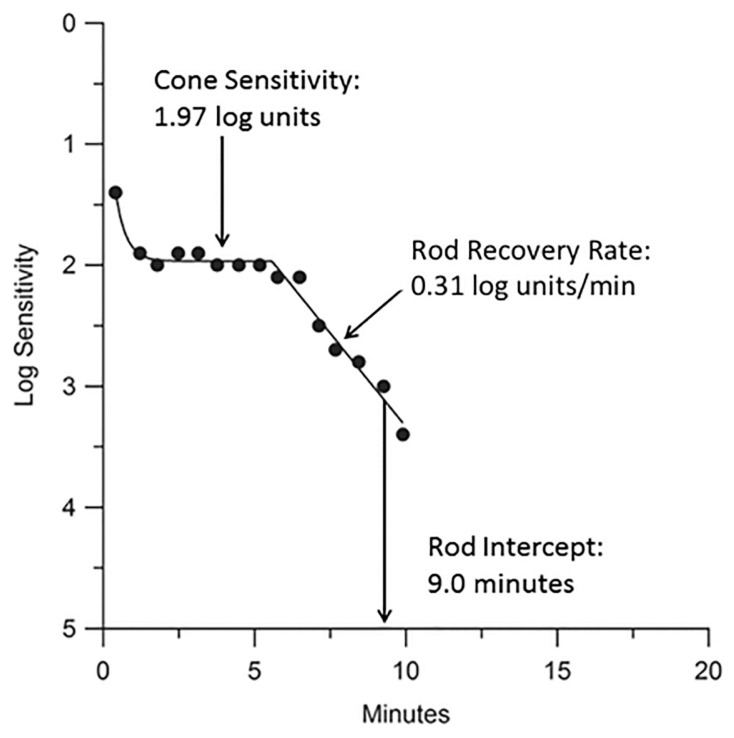
A typical DA response in a healthy 58-year-old subject using the AdaptDx. Sensitivity recovery was measured with a 2° circular test region positioned 5° superior to the fovea (black dots). An exponential–linear model was applied via nonlinear regression to fit the DA curve (line). Key parameters derived from the model include the cone plateau (1.97 log units), rod recovery rate (0.31 log units/min), and rod intercept time (RIT) (9.0 min) [9].

Dark adaptometry assesses both rod and cone sensitivity levels, as well as their respective recovery rates, offering valuable insights into the function of photoreceptors and the RPE [9]. Cone sensitivity is represented by the cone plateau phase of the DA curve, while the rod recovery rate (or rod adaptation rate) is determined by calculating the slope of the second component of the curve (Figure 2). Importantly, the rod recovery rate is independent of pupil size and magnification effects, making dark adaptometry a reliable and robust measure of retinal function [7].

A key metric in dark adaptometry testing is the rod intercept time (RIT), which represents the time required for retinal sensitivity to reach a predefined threshold of 5 × 10^−4^ cd/m^2^. The RIT is estimated through linear interpolation and is observed during the final phase of the rod recovery rate [7,9]. A shorter RIT indicates faster recovery of rod function, typically associated with normal retinal health, while a longer RIT suggests impaired DA, which may be indicative of retinal dysfunction or disease, such as age-related macular degeneration (AMD). Generally, individuals with RITs longer than 12.5 min are classified as having impaired DA, while those with RITs of 12.5 min or shorter are considered to have normal DA [7].

The rod–cone break (RCB), which occurs approximately five minutes into DA, marks the transition from cone-mediated to rod-mediated vision [6]. Sensitivity recovery during dark adaptometry is quantified by estimating the increase in sensitivity per unit of time, assuming that rod recovery follows a linear trajectory on a log-threshold, linear-time scale [2,10]. The dark adaptometry testing procedure involves presenting a stimulus light at regular intervals, with intensity adjustments based on the subject’s responses to determine the rod (scotopic) threshold. This threshold is defined as the mean sensitivity during the last five minutes of the dark adaptometry test and serves as a reliable indicator of rod function [8,11]. Collectively, these parameters allow for the precise characterization of DA kinetics and are instrumental in the early detection of retinal diseases.

### 2.3. Instruments

The primary instrument used for dark adaptometry in recent clinical studies is the AdaptDx dark adaptometer (MacuLogix, Harrisburg, PA, USA), which has gained widespread adoption due to its automated and efficient nature. Historically, the Goldmann–Weekers adaptometer was the most commonly used dark adaptometer. However, this device is no longer commercially available, and the AdaptDx has become the preferred choice over the past decade [12]. The AdaptDx is a computer-controlled system that represents a significant advancement over earlier operator-controlled devices, such as the Goldmann–Weekers. By eliminating variability introduced by manual operation, the AdaptDx enables a more consistent testing process, free from operator bias.

Furthermore, the AdaptDx offers a 20 min testing period, a significant improvement over the 40 min protocol required by the Goldmann–Weekers device [13]. This reduction in testing time helps minimize patient fatigue and enhances patient participation. Another key feature of the AdaptDx is its integration of infrared eye tracking and fixation monitoring, which ensures that the test stimulus is consistently applied to the correct retinal location [7]. This feature is particularly beneficial for patients with unstable fixation, as it ensures more accurate and reliable measurements even in the presence of eye movement during the test.

## 3. Clinical Applications of Dark Adaptometry

While long established, dark adaptometry is gaining renewed attention as a practical diagnostic tool for a wide range of retinal disorders, including inherited diseases such as RP and Stargardt’s disease, as well as acquired conditions like age-related macular degeneration (AMD), diabetic retinopathy, and vitamin A deficiency (VAD) retinopathy. Additionally, it is used to assess congenital retinal disorders and night blindness. One of the key advantages of dark adaptometry is its ability to detect early retinal dysfunction, often before any structural changes become apparent. This makes it a useful tool for early diagnosis and management in clinical practice.

### 3.1. Retinitis Pigmentosa

Retinitis pigmentosa (RP) is one of the leading causes of inherited retinal degeneration, and dark adaptometry plays a crucial role in its early detection and ongoing assessment. RP is characterized by progressive degeneration of the photoreceptors in the retina, primarily affecting the rod photoreceptors, followed by cone photoreceptor degeneration. It typically starts with night blindness (nyctalopia), whereby patients experience difficulty seeing in low-light conditions due to early rod photoreceptor dysfunction. This is often followed by prolonged DA after exposure to bright light and progressive loss of peripheral vision, commonly referred to as “tunnel vision”. As the disease advances, patients may notice photopsia (flashes of light), reduced central vision, and impaired color discrimination due to cone involvement. In later stages, individuals may experience complete loss of vision. Additional visual complaints can include light sensitivity (photophobia), poor contrast sensitivity, and increased glare. Clinical examination often reveals hallmark features such as bone spicule pigmentation in the retina, attenuated retinal vessels, and a waxy pallor of the optic disc. Functional testing, including electroretinography (ERG), typically shows reduced or extinguished rod and cone responses, while dark adaptometry reveals delayed rod-mediated recovery, often before structural changes become evident.

The molecular mechanisms underlying RP involve mutations in over 70 different genes, many of which are involved in the phototransduction pathway or the maintenance of photoreceptor structure and function. For example, pathogenic variants in the *RHO* gene, encoding the photopigment rhodopsin, are the most common cause of RP and lead to defective protein folding and rod cell death. Other pathogenic variants affect the RPE, cilium function, and retinal signaling pathways, contributing to the photoreceptor degeneration observed in RP.

A hallmark of RP is delayed rod-mediated recovery following light exposure. Dark adaptometry testing has shown that patients with RP exhibit significant abnormalities, such as increased absolute thresholds for rod function, which presents with a loss of rod system sensitivity. This impaired rod recovery can be attributed to the underlying rod photoreceptor dysfunction. A study examining RP found that patients had an abnormal increase in the rod threshold with delayed rod recovery, which is a direct consequence of the failure to regenerate rhodopsin effectively, a critical process in maintaining rod photoreceptor function.

At the molecular level, the delayed rod recovery observed in RP is linked to defective retinoid recycling, a process that is essential for photoreceptor function. In RP, pathogenic variants in genes like *RPE65*, which encodes a key enzyme in the retinoid cycle, hinder the conversion of all-trans retinal to 11-*cis* retinal, thus impeding the regeneration of rhodopsin. The inability to regenerate rhodopsin effectively leads to reduced photoreceptor sensitivity and delayed DA. Furthermore, neurodegenerative changes in the retina, including the loss of inner retinal layers and synaptic dysfunction, exacerbate the loss of rod-mediated vision. Interestingly, some studies have observed that, regardless of age, patients with RP exhibit both rod and cone adaptation abnormalities, although rod dysfunction tends to be more pronounced [14,15]. Specifically, visual sensory thresholds were increased by about four log units in all patients. This suggests that cone degeneration may occur secondarily to the primary loss of rod photoreceptors, as cones rely on the functional rod system for proper adaptation and maintenance. The cone-mediated phase of the DA curve is often impaired even in the early stages of RP, as cones suffer from secondary degenerative changes, including dysfunction in the cone–rod gap junctions and reduced metabolic support from the RPE.

As a result, dark adaptometry proves to be a valuable tool in detecting early retinal dysfunction in RP, often identifying abnormalities in rod and cone adaptation long before any noticeable impairment in visual acuity or structural changes in the retina are evident. Early detection through dark adaptometry can provide crucial information for the management and monitoring of RP patients, allowing for more informed decisions regarding potential therapeutic interventions.

### 3.2. Stargardt Disease

Stargardt disease, a form of juvenile macular degeneration, is another retinal condition in which dark adaptometry has shown clinical value. It is an inherited macular dystrophy that typically presents during adolescence with progressive, bilateral central vision loss. Clinically, patients exhibit macular atrophy along with characteristic yellow-white flecks, representing lipofuscin accumulation, distributed around the macula. Common symptoms include photophobia, color vision disturbances, and paracentral scotomas. Despite the central visual impairment, peripheral vision and rod photoreceptor function are usually preserved until the later stages of the disease, allowing many patients to retain functional night vision and mobility during the early-to-intermediate phases.

Stargardt disease is primarily caused by pathogenic variants in the *ABCA4* gene, which are responsible for over 90% of diagnosed cases. The *ABCA4* gene encodes an ATP-binding cassette transporter localized in the outer segments of photoreceptor cells, wherein it facilitates the clearance of all-trans-retinal and its derivatives from photoreceptor disc membranes. When *ABCA4* function is impaired, these retinoid by-products accumulate and undergo condensation to form toxic bisretinoid compounds, such as A2E. A2E subsequently accumulates within the RPE as part of lipofuscin granules, leading to a pathological condition known as lipofuscinosis. This process contributes to progressive RPE dysfunction and photoreceptor degeneration characteristic of Stargardt disease.

The buildup of toxic bisretinoids not only impairs the visual cycle—particularly the regeneration of 11-cis-retinal—but also induces oxidative stress within the retina. This oxidative environment, along with compromised metabolic support from the dysfunctional RPE, leads to apoptosis of photoreceptors, especially cone cells in the central macula. Over time, this cascade of degeneration underlies the progressive central vision loss that is characteristic of Stargardt disease.

Previous studies on dark adaptometry in Stargardt disease have reported mixed results. Some observed elevated cone and/or rod thresholds, while others found no abnormalities [16]. In one study involving 12 patients with Stargardt disease, rod-mediated DA appeared normal during the initial recovery phase but showed a significant delay in the later phase, particularly following the rod–cone break [16]. Specifically, the rod thresholds were elevated by 0.8 to 1.2 log units compared to normal, depending on whether the measurement was taken 35 or 60 min after the bleach. This delayed recovery may have been missed in earlier studies that reported normal outcomes, as those studies often did not include measurements of pre-bleach thresholds.

### 3.3. Age-Related Macular Degeneration

Age-related macular degeneration (AMD) is a progressive retinal disease that primarily affects central vision, with clinical manifestations and underlying mechanisms that vary across its stages. Early AMD is often asymptomatic but may present with mild metamorphopsia (distorted vision). Intermediate AMD shows drusen (subretinal deposits) and pigmentary changes. Advanced AMD includes geographic atrophy, characterized by retinal pigment epithelium (RPE) and photoreceptor loss, or choroidal neovascularization (CNV), both of which can cause severe central vision loss, scotomas, and blurred vision.

The molecular mechanisms underlying AMD pathogenesis are multifactorial and involve complement system dysregulation, oxidative stress, and chronic inflammation. Genetic variants in *complement factor H* (*CFH*), a key regulator of the alternative complement pathway, have been strongly associated with AMD susceptibility, leading to inadequate control of complement activation and subsequent inflammation. Oxidative stress, particularly from lipid peroxidation, contributes to cellular damage in the RPE and outer retina, exacerbating disease progression. Chronic inflammatory mediators such as interleukin-6 (IL-6) and tumor necrosis factor-alpha (TNF-α) further promote retinal degeneration and angiogenesis.

Drusen, a hallmark of AMD, are composed of cellular debris including amyloid-β and cholesterol, which accumulate between the RPE and Bruch’s membrane. These deposits interfere with the normal metabolic exchange between the RPE and photoreceptors, leading to functional impairment and cellular stress. In the neovascular (wet) form of advanced AMD, CNV arises due to overexpression of vascular endothelial growth factor (VEGF), a process driven by local hypoxia and inflammation. The resulting new vessels are fragile and prone to leakage, causing rapid and often severe central vision loss.

**Figure 3:**
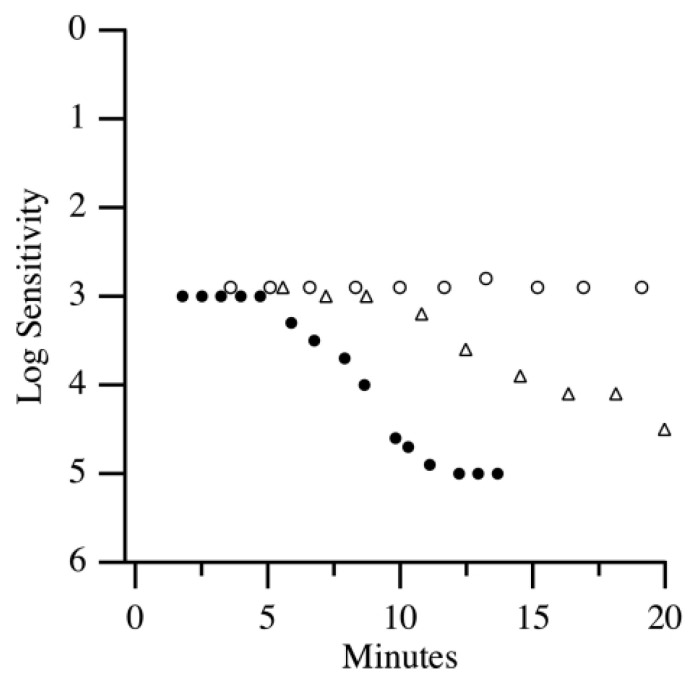
DA curves of a healthy adult (closed circles), an early AMD patient (open triangles), and an intermediate AMD patient (open circles) [7].

Dark adaptometry is increasingly recognized as a sensitive functional biomarker for the early detection of age-related macular degeneration (AMD), often revealing deficits in visual function before structural changes such as drusen become clinically apparent [17]. When assessing different stages of AMD, it was found that DA function worsens with the increasing severity of AMD. Specifically, AMD patients show substantial delays in rod-mediated recovery compared to age-matched normal adults. The mean rod intercept for AMD patients was 10 min greater than normal controls [8]. Another study also highlighted that AMD patients exhibited severe DA impairment, with the rod intercept times in AMD patients being twice as long as those in normal individuals [7]. Notably, some of the AMD patients did not reach the criterion sensitivity within the 20 min testing window and were assigned the maximum rod intercept time of 20 min, suggesting that the true functional disparity may be even greater than reported. Similar to the previously mentioned study, DA impairment increased with AMD severity (Figure 3). Both cone and rod DA parameters were also found to be valid diagnostic markers for early AMD, with significant differences in rod–cone break (RCB) ranging from 11.96 to 19.24 min between early AMD patients and controls, depending on the amount of bleach used [18]. In line with these findings, DA impairment has been shown to correlate with AMD severity (Figure 3). DA has demonstrated diagnostic value for early AMD, with significant differences observed between early AMD patients and healthy controls.

### 3.4. Diabetic Retinopathy

Diabetic retinopathy (DR) is a common microvascular complication of chronic hyperglycemia that progressively disrupts retinal structure and impairs visual function. The disease typically begins as non-proliferative diabetic retinopathy (NPDR), marked by the presence of microaneurysms, intraretinal hemorrhages, and macular edema. These early changes often manifest clinically as blurred central vision. As DR advances to proliferative diabetic retinopathy (PDR), retinal ischemia stimulates pathological neovascularization, resulting in the growth of fragile new blood vessels. These vessels are highly susceptible to rupture, leading to vitreous hemorrhage and, in severe cases, tractional retinal detachment. Patients with PDR may experience floaters, sudden vision loss, and difficulty adapting to low-light environments due to impaired DA.

At the molecular level, DR is primarily driven by chronic hyperglycemia, which induces a cascade of pathological changes in the retina. Key mechanisms include the upregulation of vascular endothelial growth factor (VEGF), accumulation of advanced glycation end-products (AGEs), and increased oxidative stress. These processes collectively contribute to endothelial dysfunction and heightened vascular permeability. A hallmark of early-stage DR is the loss of pericytes, which play a critical role in maintaining capillary stability. Pericyte degeneration is largely mediated by the activation of protein kinase C (PKC) and sustained exposure to pro-inflammatory cytokines. Their loss compromises the integrity of the blood-retina barrier, facilitating vascular leakage and the formation of microaneurysms.

Beyond vascular pathology, DR also involves significant neuronal and glial dysfunction. Retinal hypoxia, glutamate excitotoxicity, and Müller cell gliosis contribute to the progressive degeneration of retinal neurons, including photoreceptors and ganglion cells. These neurodegenerative processes play a critical role in the visual impairments reported by patients, often preceding or occurring independently of vascular changes. As such, the non-vascular components of DR are increasingly recognized as important contributors to disease progression and represent promising therapeutic targets beyond conventional anti-VEGF strategies.

DR often presents with early retinal dysfunction that precedes visible vascular abnormalities. Notably, even in the early stages of background DR, a mild form of non-proliferative diabetic retinopathy (NPDR), retinal sensitivity under scotopic conditions can be impaired, despite preserved photopic vision [19]. Dark adaptometry has revealed elevated rod thresholds in these patients, indicating early rod system dysfunction. This was further supported by Bavinger et al. [9], who demonstrated significantly delayed rod recovery in individuals with moderate NPDR. Their findings suggest that rod photoreceptor dysfunction occurs early in the disease course, well before the development of proliferative diabetic retinopathy (PDR). In contrast, cone sensitivity appears to remain relatively preserved until later stages, with significant impairment observed primarily during PDR. Given that DA increases the retina’s demand for oxygen and glucose, these functional deficits may reflect broader impairments in retinal energy metabolism associated with diabetic retinopathy [1].

### 3.5. Cone–Rod Dystrophy

Dark adaptometry is also a valuable diagnostic tool in cone–rod dystrophies (CRD), a group of inherited retinal degenerations characterized by early and progressive cone photoreceptor dysfunction, followed by rod involvement. Patients typically present in childhood or adolescence with symptoms such as photophobia, decreased color discrimination, and central vision loss that is often perceived as central scotomas. In advanced stages, fundus examination may reveal macular atrophy and bone spicule-like pigment deposits. While these retinal changes resemble those seen in RP, CRD is distinguished by its earlier cone involvement and differing pattern of disease progression.

At the molecular level, CRD is genetically heterogeneous, with pathogenic variants identified in multiple genes essential for photoreceptor development and function. These include *GUCY2D*, which encodes retinal guanylate cyclase critical for cyclic GMP (cGMP) synthesis; *CRX*, a transcription factor vital for photoreceptor gene regulation; and *PDE6C*, which encodes a cone-specific phosphodiesterase required for cGMP hydrolysis during phototransduction. Mutations in these genes disrupt phototransduction cascades, impair visual signaling, and trigger photoreceptor apoptosis.

In CRD, primary cone dysfunction is followed by secondary rod degeneration, which is thought to result from disrupted metabolic and structural interactions between cones and rods, along with impaired support from the RPE. The loss of trophic signaling between photoreceptors and the RPE accelerates outer retinal degeneration and contributes to the progressive decline in visual function observed in affected individuals.

One study reported that patients with CRD exhibited an abnormal, nearly monophasic DA curve, reflecting absent or severely diminished cone function. Although the rate of rod threshold recovery may appear relatively normal, the final rod thresholds are elevated, indicating early rod dysfunction [20]. This altered response pattern is consistent with the progressive nature of CRD, whereby cone degeneration occurs first and is followed by secondary rod involvement. Supporting this, Gregory-Evans et al. (2000) reported similar findings, noting progressive visual field loss during DA testing in CRD patients, particularly in both central and peripheral regions [21].

### 3.6. Vitamin A Deficiency Retinopathy

Vitamin A deficiency (VAD) retinopathy is a progressive ocular disorder caused by insufficient levels of retinol, an essential component of the visual cycle. Clinically, the condition typically presents first with nyctalopia, as rod photoreceptors are highly dependent on vitamin A for proper function. Additional ocular signs may include conjunctival xerosis and Bitot’s spots, which are foamy keratinized deposits on the conjunctiva. If the deficiency persists, it can progress to xerophthalmia, marked by corneal dryness, ulceration, and ultimately irreversible blindness in severe or untreated cases.

At the molecular level, VAD retinopathy results from disruption of the visual cycle. Vitamin A (retinol) is critical for the regeneration of 11-*cis*-retinal, the chromophore required for forming rhodopsin, the light-sensitive pigment in rod cells. In the absence of adequate retinol, 11-*cis*-retinal synthesis is impaired, leading to reduced rhodopsin availability and halted phototransduction in rod photoreceptors. Because rods have a particularly high demand for retinoids, they are the first to be affected and degenerate in VAD.

In some cases, VAD arises from impaired transport mechanisms. This can occur due to pathogenic variants in the gene encoding retinol-binding protein (RBP), which is responsible for transporting vitamin A from hepatic stores to peripheral tissues, including the retina. In addition, conditions such as malnutrition, fat malabsorption syndromes, and liver disease can hinder retinoid mobilization and storage, compounding the risk of retinal degeneration.

VAD retinopathy is closely associated with abnormal DA, reflecting the critical role of vitamin A in photoreceptor function. Renner et al. (2015) reported a VAD patient with absent rod function but preserved cone function, particularly for green and red stimuli, suggesting selective rod involvement in VAD [22]. This was confirmed using two-color DA, which revealed depressed thresholds in both rods and cones, indicating more advanced retinal dysfunction [23]. Seeliger et al. (1999) further confirmed these findings by observing elevated DA thresholds of one log unit above the normal cone threshold and a complete absence of rod function in two VAD patients [24]. Collectively, these studies underscore the sensitivity of dark adaptometry in detecting early functional deficits in patients with VAD.

### 3.7. Congenital Stationary Night Blindness

Congenital stationary night blindness (CSNB) is a non-progressive retinal disorder characterized by lifelong nyctalopia that typically begins in early childhood. Unlike degenerative retinal diseases, CSNB remains stable over time. In addition to night blindness, patients may present with nystagmus, myopia, and reduced visual acuity, particularly under scotopic conditions. Electroretinography (ERG) is essential for diagnosis and typically reveals absent or markedly reduced rod responses, along with abnormal cone-mediated signaling depending on the CSNB subtype.

At the molecular level, CSNB is primarily caused by defects in synaptic transmission between photoreceptors and bipolar cells, which most commonly affects the ON-bipolar cell pathway. However, some forms of CSNB result from presynaptic defects that disrupt neurotransmitter release from photoreceptors, impairing signal transmission to both ON- and OFF-bipolar cells. CSNB is broadly classified into two subtypes: the complete form (cCSNB), which involves a selective dysfunction of the ON-pathway, and the incomplete form (incCSNB), which affects both ON- and OFF-pathways due to abnormalities in photoreceptor synaptic output.

Pathogenic variants in genes such as *NYX* (encoding nyctalopin) and *GRM6* (encoding a metabotropic glutamate receptor) disrupt the ability of ON-bipolar cells to depolarize in response to rod-mediated glutamatergic signaling. These genes are commonly associated with the complete form of congenital stationary night blindness (cCSNB), which selectively affects the ON-bipolar cell pathway. Another frequently implicated gene, *CACNA1F*, encodes a voltage-gated calcium channel essential for neurotransmitter release from photoreceptors and is typically linked to incomplete CSNB (incCSNB). These molecular disruptions underlie the characteristic electroretinographic (ERG) phenotypes seen in CSNB and are central to its clinical manifestation.

CSNB can be effectively evaluated using DA. Although previous studies have reported varying results, all consistently demonstrate significant abnormalities in patients with CSNB. Bech-Hansen et al. (1998) observed that both cCSNB and incCSNB exhibit elevated rod thresholds, though with differing degrees of severity, which highlights the heterogeneous nature of the disorder [25]. In another study, X-linked cCSNB was associated with an elevated cone threshold, while incCSNB showed a more prominent elevation in rod thresholds, ranging from 1.0 to 1.5 log units during dark adaptometry [26]. Similarly, two studies reported absent rod-mediated DA and elevated cone thresholds in individuals with night blindness, further emphasizing the disrupted rod–cone interaction characteristic of CSNB [27,28].

## 4. Limitations and Challenges

While dark adaptometry offers a unique advantage by directly assessing photoreceptor function, especially in the early stages of retinal disease, it is important to consider its clinical utility alongside other established imaging modalities. Optical coherence tomography (OCT) and fundus autofluorescence (FAF), for example, are widely used structural imaging techniques that can detect early morphological changes, such as subretinal deposits and RPE abnormalities, often before they become clinically visible on ophthalmoscopy. While dark adaptometry may detect dysfunction prior to observable structural damage, this functional insight currently does not alter clinical management in some cases, particularly due to the lack of available therapies at those early stages. Furthermore, dark adaptometry requires longer testing times and greater patient cooperation compared to faster imaging, like OCT or FAF. Therefore, while dark adaptometry provides valuable complementary information about retinal physiology, its adoption must be weighed against practical considerations, such as examination time, staffing, and patient throughput in modern ophthalmic clinics.

**Figure 4:**
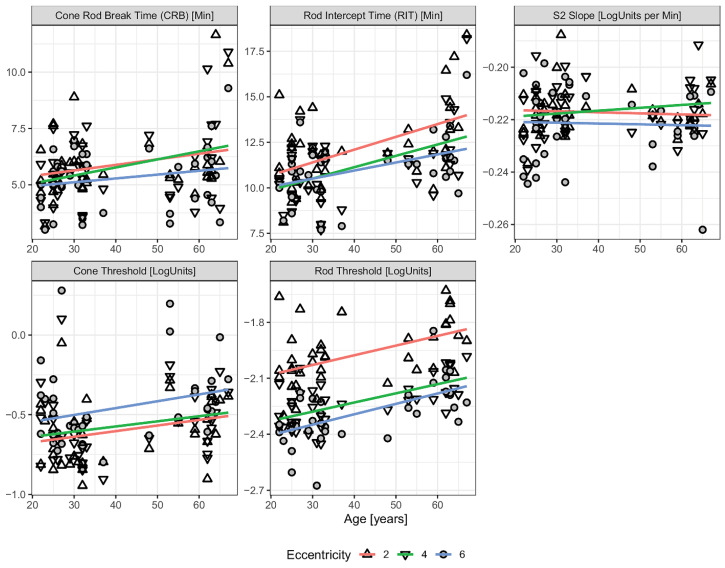
DA parameters as a function of age across different retinal eccentricities. Eccentricity is denoted by different data point shapes. Single linear regression lines are fitted independently for each eccentricity. The parallel trends across all three tested locations indicate no significant interaction between eccentricity and age, suggesting that age-related changes in DA occur uniformly across the retina [29].

Although dark adaptometry is valuable for assessing photoreceptor recovery and early retinal dysfunction, such as AMD, it faces several limitations that can affect its reliability and broader clinical application. Factors such as media opacities and the need for patient cooperation can complicate data interpretation and hinder large-scale implementation in clinical trials. One key challenge is inter-individual variability, as patient demographics, comorbidities, and ocular media clarity can all influence test outcomes. Inconsistencies in testing methods further reduce comparability across studies. In a study evaluating age-related changes in DA parameters, rod intercept time, cone threshold, and rod threshold were all found to increase with age (Figure 4) [29]. Other investigations have similarly demonstrated that rod-mediated DA slows with age, as reflected by a decline in the slope of second linear portion of the rod-mediated DA curve (S2 slope) (approximately 0.01 log units per decade) and an increase in rod intercept time (RIT) (by about 0.5 min per decade)—both indicative of reduced rhodopsin regeneration in older populations [9,30,31]. Notably, elderly individuals, particularly those with AMD, exhibit greater variability in dark adaptometry results compared to younger subjects. This makes it more difficult to distinguish disease-related functional decline from normal age-associated changes, further underscoring the need for improved standardization and age-adjusted normal ranges in dark adaptometry [30].

Participant cooperation is another notable challenge in DA. The extended testing time required for accurate data collection often leads to patient fatigue, noncompliance, or withdrawal from participation [18]. Traditional methods such as the Goldmann–Weekers adaptometer, which typically require 40–60 min of continuous assessment, are particularly susceptible to variability due to declining patient attention and stamina [13]. Although newer devices like AdaptDx and updated protocols have reduced testing times to 20–40 min, the duration still poses a barrier to full cooperation. Fatigue, delayed responses, or lapses in attention can introduce significant noise into sensitivity measurements, particularly in elderly individuals or patients with cognitive or attentional limitations [8,32,33]. These factors may result in erratic or incomplete threshold curves, undermining the reliability of the test. To mitigate these issues, clear procedural guidelines and strict quality control measures are essential to maintain data accuracy and reproducibility.

Age-related changes in ocular media, such as reduced pupillary size and lens transparency, can influence DA measurements. While nuclear sclerosis, a common form of cataract, minimally affects visual acuity, studies suggest that lens opacities alone do not fully account for delayed DA in aging populations [32]. However, other media abnormalities, such as corneal opacities, vitreous haze, or any advanced alterations in ocular clarity, remain understudied and may interfere with retinal light exposure during testing. These potential confounders emphasize the importance of evaluating ocular media status alongside DA assessments to ensure that observed deficits are due to true retinal dysfunction rather than pre-retinal optical factors. To mitigate this, assessments of media clarity, such as slit-lamp examination or Scheimpflug imaging, should be conducted prior to DA testing. Moreover, integrating objective metrics such as directional or diffuse fundus reflectometry could help normalize light delivery by quantifying backscatter and absorption characteristics of the retina [34].

Systemic conditions, such as membranoproliferative glomerulonephritis type II (MPGN II), may indirectly impair DA through structural alterations in Bruch’s membrane. It is hypothesized that the electron-dense deposits in Bruch’s membrane of MPGN II disrupt the metabolic exchange between the retina and choroid, thereby compromising photoreceptor function and delaying retinal adaptation. Similar pathogenic mechanisms have been proposed in Sorsby fundus dystrophy and AMD, wherein barriers to nutrient diffusion across Bruch’s membrane are believed to contribute to retinal dysfunction [35]. These examples underscore the complexity of differentiating primary retinal disease from the secondary effects of systemic conditions. To avoid misdiagnosis and ensure accurate interpretation of functional tests like dark adaptometry, a comprehensive clinical evaluation, including systemic and ocular assessments, is essential.

The lack of standardized protocols and equipment remains a significant barrier to the integration of dark adaptometry into routine clinical practice [18]. Most studies rely on a specific device without consistent benchmarks or interoperability across systems. Variability in stimulus parameters, luminance, diagnostic thresholds, retinal eccentricity, and testing protocols hinders cross-study comparisons and contributes to inconsistent findings due to differing methodological limitations [8]. Establishing standardized calibration protocols and reference values would improve the reliability and cross-study utility of dark adaptometry. Furthermore, testing in artificial, highly controlled environments may overestimate subclinical deficits, as these conditions fail to reflect real-world visual demands and ambient light variability [5]. To enhance the clinical utility of DA, it is crucial to strike a balance between diagnostic accuracy and practical feasibility. This may involve streamlining protocols and reducing test duration without compromising sensitivity or reliability, thereby improving patient compliance and facilitating broader clinical adoption.

While early clinical applications of dark adaptometry have currently focused on a few diseases, this represents only an initial step. Given that dark adaptometry assesses a fundamental aspect of visual function, broadening its validation across diverse retinal and systemic diseases is essential to support its wider clinical adoption.

## 5. Conclusions

Dark adaptometry is a powerful diagnostic tool that offers critical insights into retinal function and disease progression. By evaluating the time course of photoreceptor recovery following light exposure, it facilitates the early detection of retinal disorders such as AMD, RP, and DR, often before structural abnormalities are clinically observable (Table 1). Its ability to distinguish between rod and cone recovery kinetics makes it especially valuable for phenotyping retinal disease and monitoring progression. Despite its clinical promise, dark adaptometry faces several challenges, including inter-individual variability, lengthy testing protocols, and a lack of standardization across devices and methodologies. Overcoming these barriers through refined protocols, streamlined testing platforms, and technological advancements will enhance both its accessibility and diagnostic precision. As ongoing research continues to optimize dark adaptometry assessment, its role in early intervention, disease monitoring, and patient care is expected to grow significantly within ophthalmic practice. As one of several multimodal tests, including OCT and FAF, dark adaptometry provides complementary functional insights that can be integrated into clinical decision-making.

## Figures and Tables

**Table 1:** Summary of changes in rod- and cone-mediated DA in retinal disorders.

Disease	Primary DA Abnormality	Key DA Metrics	Clinical Utility	Photoreceptor Dominance	DA Deficit Timing
Retinitis Pigmentosa (RP)	Delayed rod- and cone-mediated recovery	↑ Rod threshold and delayed rod–cone break	Early rod and cone dysfunction detection	Rod > Cone	Precedes structural changes
Stargardt Disease	Delayed rod recovery after rod–cone break	↑ Rod and/or cone threshold	Detection of late-phase rod dysfunction	Rod = Cone (early); Rod > Cone (late)	Follows structural changes
Age-Related Macular Degeneration (AMD)	Abnormal rod and cone adaptation	↑ Rod intercept time (RIT) and delayed rod threshold	Sensitive marker for early AMD and correlates with AMD severity	Rod > Cone	Precedes structural changes
Diabetic Retinopathy (DR)	Delayed rod-mediated recovery; early rod impairment; and late cone impairment	↑ Rod threshold	Detection of early retinal dysfunction before vascular signs	Rod > Cone (early); Cone > Rod (late)	Precedes structural changes
Cone–Rod Dystrophy (CRD)	Diminished rod and cone function	↑ Rod threshold	Monitoring of sequential photoreceptor degeneration	Cone > Rod	Follows structural changes
Vitamin A Deficiency (VAD)	Absent or delayed rod function	↑ Rod and cone thresholds	Early detection of rod dysfunction	Rod > Cone	Precedes structural changes
Congenital Stationary Night Blindness (CSNB)	Absent or delayed rod function	↑ Rod and cone thresholds	Confirmation of dysfunctional rod–cone signaling and support of subtype differentiation	Rod > Cone	Precedes structural changes

↑: Increase.

## Data Availability

Data are contained within the article.
